# Outcome Prediction of Acute Kidney Injury Biomarkers at Initiation of Dialysis in Critical Units

**DOI:** 10.3390/jcm7080202

**Published:** 2018-08-06

**Authors:** Vin-Cent Wu, Chih-Chung Shiao, Nai-Hsin Chi, Chih-Hsien Wang, Shih-Chieh Jeff Chueh, Hung-Hsiang Liou, Herbert D. Spapen, Patrick M. Honore, Tzong-Shinn Chu

**Affiliations:** 1 Division of Nephrology, National Taiwan University Hospital, No. 7 Chung-Shan South Road, Zhong-Zheng District, Taipei 100, Taiwan; q91421028@ntu.edu.tw; 2 Division of Nephrology, Department of Internal Medicine, Saint Mary’s Hospital Luodong, No. 160 Chong-Cheng South Road, Loudong, Yilan 265, Taiwan; chungyy2001@yahoo.com.tw; 3 Saint Mary’s Junior College of Medicine, Nursing and Management College, No. 100, Ln. 265, Sec. 2, Sanxing Rd., Sanxing Township, Yilan 266, Taiwan; 4 Surgery Department, National Taiwan University Hospital, No. 7 Chung-Shan South Road, Zhong-Zheng District, Taipei 100, Taiwan; chinaihsin@ntuh.gov.tw (N.-H.C.); wchemail@gmail.com (C.-H.W.); 5 Glickman Urological and Kidney Institute, Cleveland Clinic Lerner College of Medicine, Cleveland Clinic, Cleveland, 9500 Euclid Ave., Cleveland, OH 44195, USA; jeffchueh@gmail.com; 6 Division of Nephrology, Department of Internal Medicine, Hsin-Jen Hospital, Dialysis Center, Hsin-Ren Clinics, No. 395, Chung-Shan Road, New Taipei City 231, Taiwan; hh258527@ms23.hinet.net; 7 ICU Department, Universitair Ziekenhuis Brussel, Vrije Universiteit Brussel, 101, Laarbeeklaan, 1090 Jette, Belgium; 8 ICU Department, CHU Brugmann University Hospital, 4 Place Arthur Van Gehucthen, 1020 Brussels, Belgium; 9 NSARF Group (National Taiwan University Hospital Study Group of ARF), Taipei 100, Taiwan

**Keywords:** acute kidney injury, biomarker, fibroblast growth factor-23, kidney injury molecule-1, mortality, neutrophil gelatinase-associated lipocalin, renal replacement therapy

## Abstract

The ideal circumstances for whether and when to start RRT remain unclear. The outcome predictive ability of acute kidney injury (AKI) biomarkers measuring at dialysis initializing need more validation. This prospective, multi-center observational cohort study enrolled 257 patients with AKI undergoing renal replacement therapy (RRT) shortly after admission. At the start of RRT, blood and urine samples were collected for relevant biomarker measurement. RRT dependence and all-cause mortality were recorded up to 90 days after discharge. Areas under the receiver operator characteristic (AUROC) curves and a multivariate generalized additive model were applied to predict outcomes. One hundred and thirty-five (52.5%) patients died within 90 days of hospital discharge. Plasma c-terminal FGF-23 (cFGF-23) had the best discriminative ability (AUROC, 0.687) as compared with intact FGF-23 (iFGF-23) (AUROC, 0.504), creatinine-adjusted urine neutrophil gelatinase-associated lipocalin (AUROC, 0.599), and adjusted urine cFGF-23 (AUROC, 0.653) regardless whether patients were alive or not on day 90. Plasma cFGF-23 levels above 2050 RU/mL were independently associated with higher 90-day mortality (HR 1.76, *p* = 0.020). Higher cFGF-23 levels predicted less weaning from dialysis in survivors (HR, 0.62, *p* = 0.032), taking mortality as a competing risk. Adding cFGF-23 measurement to the AKI risk predicting score significantly improved risk stratification and 90-day mortality prediction (total net reclassification improvement = 0.148; *p* = 0.002). In patients with AKI who required RRT, increased plasma cFGF-23 levels correlated with higher 90-day overall mortality after discharge and predicted worse kidney recovery in survivors. When coupled to the AKI risk predicting score, cFGF-23 significantly improved mortality risk prediction. This observation adds evidence that cFGF-23 could be used as an optimal timing biomarker to initiate RRT.

## 1. Background

Renal replacement therapy (RRT) is life-saving in patients with acute kidney injury (AKI) but is not devoid of serious complications and severe adverse events [1]. Patients who, even temporarily, require RRT also may develop more frequently long-term or end-stage renal disease (ESRD) and have a higher mortality risk [2]. The need for and the optimal timing to initiate RRT are crucial yet unresolved issues [1,3].

Nephrologists continuously look out for kidney specific biomarkers that assist in fine-tuning of diagnosis, treatment, and prognosis of AKI [4]. Few biomarkers were validated as outcome-specific biomarkers in critically ill patients at initiation of RRT. Urine neutrophil gelatinase-associated lipocalin (NGAL) was one of the first biomarkers to be validated for predicting short-term mortality in patients with advanced AKI [5] and recently became part of the indicators to decide early start of dialysis [6]. Interleukin-18 (IL-18) at the commencement of dialysis could also predict hospital mortality in critically ill patients [7]. Adding plasma interleukin-8 to a parsimonious clinical model (i.e., age, mean arterial pressure, mechanical ventilation, and bilirubin) augmented prediction of renal recovery and AKI mortality compared with using only the clinical variables [8].

Fibroblast growth factor 23 (FGF-23), a peptide initially recognized for its phosphaturic role in rare genetic or acquired hypophosphatemia disorders [9], is one of the most recently proposed kidney biomarkers. FGF-23 acts as a hormone that significantly influences phosphate, vitamin D, and bone mineral homeostasis [10]. Several research groups have proposed cFGF-23 as a biomarker for predicting early occurrence of AKI, evaluating prognosis of chronic kidney disease (CKD), and estimating cardiovascular morbidity and mortality [11,12,13,14,15].

An important area of AKI research particularly focuses on reinforcing current dialysis requiring AKI by adding measurement of (a) sensitive biomarker (s) to assess the impact of RRT on relevant patient outcome variables. Within this perspective, we designed a study to evaluate the predictive capacity of various structural and functional kidney biomarkers (including the novel markers cFGF-23 and iFGF-23) and disease severity scores, measured at initiation of RRT, on survival and renal function recovery in a cohort of AKI patients.

## 2. Methods

### 2.1. Registration of Clinical Trials

This study was approved by the University’s Institutional Review Board (201409024RINB in National Taiwan University Hospital, 01-X16-059 in Buddhist Tzu Chi General Hospital, and TYGH104007 in Taoyuan General Hospital) and written informed consent was obtained from all subjects participating in the trial. The trial was registered prior to patient enrollment at clinicaltrials.gov (NCT01503710, Principal investigator: V.-C.W, Date of registration: 28 February 2012).

### 2.2. Study Population

The study was conducted by the National Taiwan University Study Group on Acute Renal Failure (NSARF) and based on a prospectively created AKI database [16,17,18,19,20]. From August 2011 until January 2015, 257 AKI patients who required RRT after intensive care unit (ICU) admission were prospectively enrolled. Exclusion criteria included: age <18 years, previous nephrectomy, renal transplantation or RRT treatment, ICU or hospital length of stay of respectively <2 days and >180 days during the index hospitalization, and AKI caused by urologic surgery induced injury, vasculitis, obstruction, glomerulonephritis, interstitial nephritis, hemolytic uremic syndrome, or thrombotic thrombocytopenic purpura.

### 2.3. Data Collection

Baseline characteristics, including demographic data, co-morbidities, the cause of AKI. For the risk prediction before initializing dialysis, the individual AKI risk predicting score was calculated [21]. The worst physiological values and biochemical data on the index day were recorded.

Baseline serum creatinine (sCr) was the nadir value obtained after the previous admission in those who had more than one admission within 1 year before the index admission, or estimated with the Modification of Diet in Renal Disease equation (assuming an average eGFR of 75 mL/min/1.73 m^2^) [22]. Peak sCr was defined as the highest sCr before RRT initiation in the ICU. Indication for dialysis and organ failure were defined as previously reported [16,23,24] (Supplemental Data file).

RRT modalities in each patient were initially chosen by the attending physician and adapted according to hemodynamic evaluation and evolution by the critical care nephrologist (Supplemental Data file).

### 2.4. Measurements of Kidney Biomarkers

The urine samples, collected in separate polypropylene tubes containing sodium azide at dialysis initiation, were stored at −80 °C until required. Each specimen was centrifuged (800× *g* at 4 °C for 5 min) and the supernatant was collected for ELISA analysis.

Kidney biomarker levels were assessed with a human FGF-23 C-terminal/intact-terminal ELISA kit (Immutopics; San Clemente, CA, USA), a human KIM-1, and a lipocalin-2/NGAL ELISA kit (R&D Systems, Inc., Minneapolis, MN, USA).

The cFGF-23 and iFGF-23 values were expressed in relative units (RU)/mL and pg/mL, respectively. The coefficient of variation was 4.4% for iFGF-23 and 4.0% for cFGF-23. The lower limits for detection of cFGF-23, iFGF-23, KIM-1 and NGAL were 0.156 RU/mL, 0.2 pg/mL, 0.046 ng/mL, and 0.04 ng/mL, respectively were completed as described by the manufacturer’s protocol and performed in duplicate. 1, 25 di hydroxyvitamin D was measured using DiaSorin radioimmunoassay assays kit (Stillwater, MN, USA) and total 25-hydroxyvitamin D was measured using an electro-chemiluminescence (Elecsys^®^ Vitamin D total, Cobas, Roche©). Urine creatinine levels were measured with the Jaffe assay, with standardization of the isotope dilution mass spectrometry traceable reference.

### 2.5. Outcome Definitions

Primary clinical endpoints were 90-day mortality after hospital discharge and dialysis dependency at 90 days in survivors. Secondary end-points included a 90-day composite outcome (ongoing dialysis or 90-day mortality after discharge), in-hospital mortality, and a composite outcome at discharge (ongoing dialysis or mortality at discharge). All patients were followed until death or for a time span exceeding 90 days after discharge, whichever occurred first. Successful withdrawal from dialysis was defined as surviving without dialysis at the end of study.

### 2.6. Statistical Analysis

All the univariate significant and non-significant relevant covariates, including age, sex, baseline comorbidities, indication for dialysis, etiology of AKI, kidney function profile (e.g., baseline eGFR and candidate biomarkers, candidate biomarkers and SOFA score at dialysis initiation, dialysis modality, and some of their interactions were put on the variable lists to be selected (Table 1). Two-sample student’s *t*-test was used to analyze continuous data and χ^2^ test or Fisher’s exact test was used to analyze categorical data. The accumulated hazard ratio was modeled by Cox regression models and adjusted for the covariates for the outcomes of interest (Supplemental Data file). The significance levels for entry (SLE) and for stay (SLS) were set to 0.15 for being conservative. Then, with the aid of substantive knowledge, the best candidate final logistic regression model was identified manually by dropping the covariates with *p* > 0.05 one at a time until all regression coefficients were significantly different from 0.

Area under the receiver operating characteristic (AUROC) curves were generated to evaluate biomarker performance. We use the methods of Hanley & McNeil (PMID, 6878708) for the calculation of the Standard Error of the Area Under the Curve (AUC) and of the difference between two AUCs. A generalized additive model (GAM) (with spline), incorporating the subject-specific (longitudinal) random effects, was plotted with adjustment for other clinical parameters to assess outcome-predictive effects of candidate biomarkers in individual patients [25,26].

Nonlinear effects of continuous covariates were explored with simple and multiple GAMs, which determine appropriate cut-off point(s) for discriminating candidate biomarkers, if necessary, during the stepwise variable selection procedure. The optimal cut-off value was defined as the log odd equaling zero [27].

Because of the high mortality rate among dialysis patients, competing-risk regression using the Fine and Gray model by considering the subdistribution hazard was also performed [28].

Net re-classification improvement (NRI) and integrated discrimination improvement (IDI) were used to evaluate the ability of candidate biomarkers for more accurate stratification of individuals into higher or lower risk categories (re-classification). Regarding 90-day mortality, an increase in NRI was calculated in a model containing both the AKI risk predicting score [21] and the cFGF-23 measurements, and the result was compared with the AKI risk predicting score alone. We defined 0–20%, 20–80%, and >80% as risk categories and re-classified patients with mortality by decision curve analysis and scatter plot (Supplemental Data file). A *p* < 0.05 was considered significant.

## 3. Results

### 3.1. Clinical Characteristics

Two hundred and fifty-seven patients (mean age 65.7 years; 167 (65%) male) with AKI who required RRT were studied. Average SOFA, APACHE II, and MODS scores were respectively 8.9, 16.3, and 5.9.

The main causes of AKI were shock (58.4%), sepsis (38.1%) and contrast nephrotoxicity (14.4%). Nine patients (3.5%) had stage 1 AKI, 58 (22.6%) patients had stage 2 AKI, and 190 (73.9%) patients had stage 3 AKI at RRT initiation. The most frequent indication for RRT was oliguria (64.6%), followed by azotemia (47.9%) and fluid overload (43.2%) (Table 1).

### 3.2. Hospital and 90-Day Outcomes

The in-hospital mortality rate, composite outcome at discharge, 90-day mortality rate and 90-day composite outcome rate were respectively 48.2%, 67.3%, 52.5%, and 70.4%. Table 1 shows baseline characteristics, pre-RRT and outcome parameters of patients categorized by 90-day mortality and 90-day composite outcome, respectively. Patients who did not survive at 90 days or with a 90–day composite outcome were older, had lower urine output, higher disease severity, risk predicting scores and received higher doses of inotropic equivalents than survivals (Table 1).

Importantly, only higher plasma cFGF-23 levels enabled to differentiate patients with both 90-day mortality/composite outcomes from those without events (*p* = 0.001).

### 3.3. Discriminative Power of Biomarkers for 90-Day Relevant Outcomes

**Figure 1:**
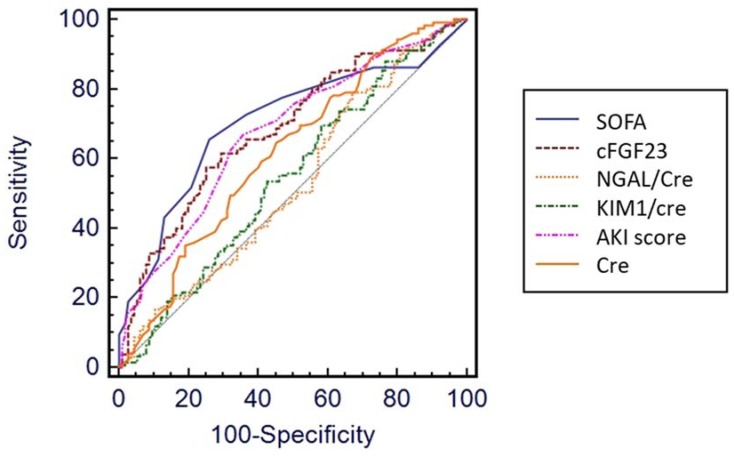
Comparisons of predictive powers for 90-day mortality among different variables. Note: the comparison was performed using the area under the receiver operator characteristic curves (AUROCs). Abbreviations: Cre, creatinine; cFGF-23, c-terminal fibroblast growth factor-23; KIM-1, Kidney Injury Molecule-1; NGAL, neutrophil gelatinase-associated lipocalin; SOFA, Sequential Organ Failure Assessment; KDIGO, Kidney Disease Improving Global Outcomes; AKI, acute kidney injury.

Levels of SOFA (AUROC, 0.706), AKI risk predicting score (0.677), sCr (0.619), cFGF-23 (0.687), plasma iFGF-23 (0.504), creatinine-adjusted urine NGAL (0.599), adjusted urine cFGF-23 (0.653) and adjusted urine KIM-1 (0.547) at initiation of dialysis could predict 90-day mortality. Plasma cFGF-23 demonstrated better discriminative ability than NGAL for mortality at 90 days (*p* = 0.001 by AUROC comparison) (Figure 1, *p* at Table S1).

**Figure 2:**
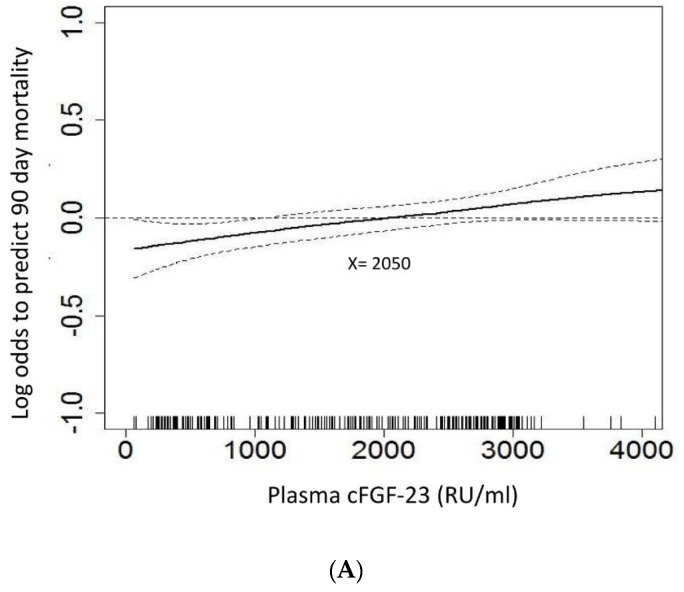

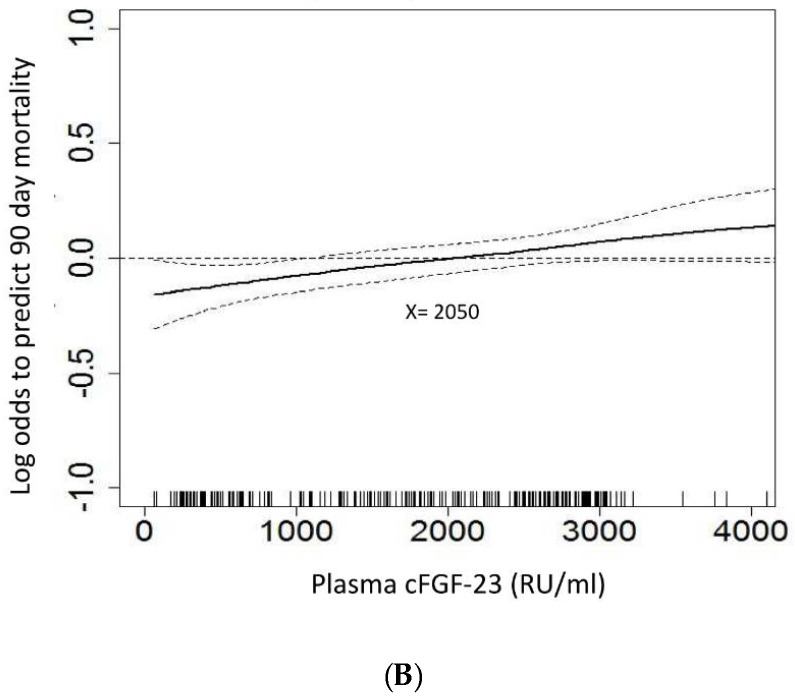
Generalized additive model (GAM) plot for the probability of (**A**) 90-day mortality, and (**B**) 90-day composite outcome against serum cFGF-23 levels at initiation of dialysis. Note: The GAM plot was incorporated with the subject-specific (longitudinal) random effects expressed as the logarithm of the odds (logit). The probability of outcome events was constructed with cFGF-23 levels averaging zero over the range of the data, i.e., cFGF-23 = 2050 ng/mL. All the relevant covariates, including characteristics, comorbidities, laboratory data, at intensive care unit (ICU) admission, etiology of acute kidney injury (AKI), indication for dialysis, dialysis modality, SOFA score, and plasma cFGF-23 at dialysis, and some of their interactions, such as interventions listed in Table 1, were put on a selected variable list to predict the outcome of interest.

The GAM plot showed a positive correlation between increased plasma cFGF-23 levels at start of dialysis and the log of the odds of 90-day mortality and composite outcome. After adjusting all variables listed in Table 1 for nonlinear effects, plasma cFGF-23, at a cut-off value of 2050 RU/mL by the GAM model, demonstrated independently good prediction of both 90-day mortality (Figure 2A) and 90-day composite outcome (Figure 2B).

### 3.4. Plasma cFGF-23 and Outcome

Using a cut-off value of 2050 RU/mL, patients were divided in a “high” and a “low” cFGF-23 group. Subjects with high cFGF-23 had lower baseline sCr, but higher phosphate concentrations at dialysis initiation, higher in-hospital and 90-day mortality, lower dialysis weaning rate and higher composite outcome results (Table 2).

A high cFGF-23 level represented an independent risk factor for in-hospital mortality (OR, 1.80, *p* = 0.049), composite outcome at discharge (OR, 1.80, 95% CI = 1.01–3.24; *p* = 0.043), 90–day mortality (OR, 2.19, 95% CI = 1.20–4.00; *p* = 0.011), and 90-day composite outcome (OR, 2.39, 95% CI = 1.31–4.35; *p* = 0.005) after adjusting for age, gender, baseline eGFR, and factor interaction with cFGF-23 and SOFA score. Importantly, no interaction was observed between the cFGF-23 level and underlying diabetes mellitus, baseline eGFR, age, and AKI risk predicting score at dialysis initiation (all *p* > 0.05) (Table 3).

**Figure 3:**
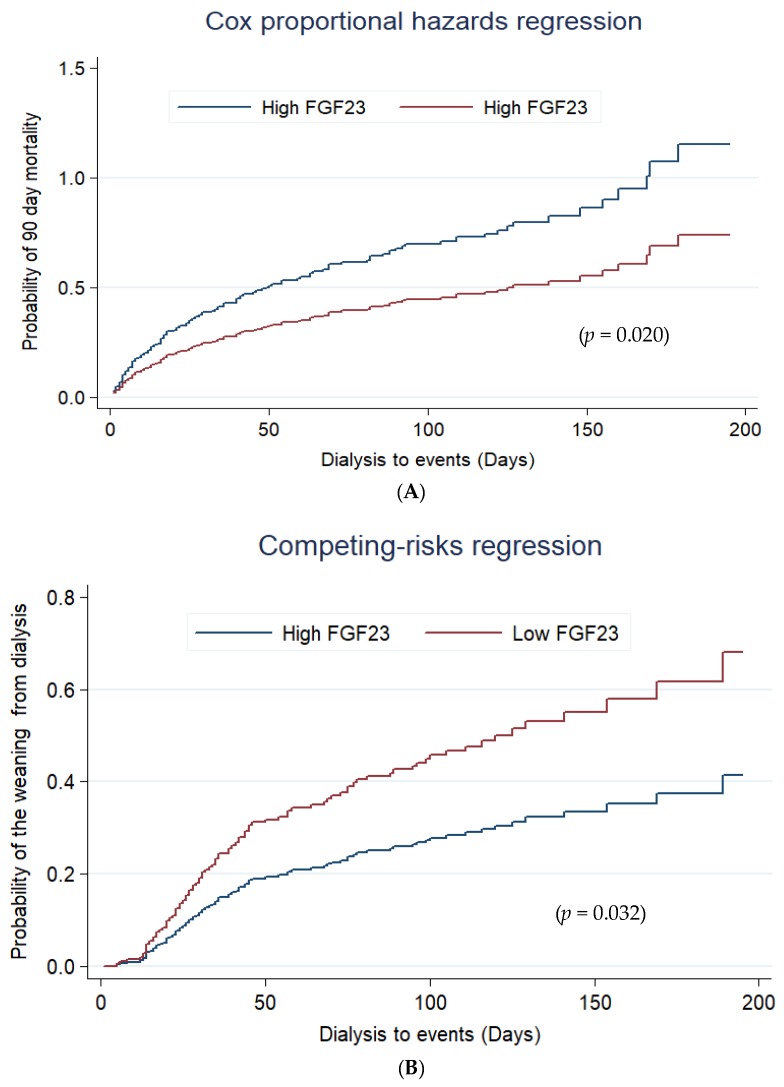
Cox proportional hazard plots stratified by serum cFGF-23 level for assessing probability of 90-day mortality (**A**) and the weaning from dialysis (**B**) by competing analysis and with mortality as a risk factor. Abbreviations: cFGF-23, c-terminal fibroblast growth factor-23; Using a cut-off value of 2050 RU/mL of cFGF-23 at initializing dialysis, patients were divided in a “high” and a “low” cFGF-23 group; all the relevant covariates, including characteristics, comorbidities, laboratory data, at ICU admission, etiology of AKI, indication for dialysis, dialysis modality, SOFA score, and plasma cFGF-23 at dialysis, and some of their interactions, such as interventions listed in Table 1, were put on a selected variable list to predict the outcome of interest.

Cox proportional hazard regression analysis revealed that patients undergoing RRT who displayed high cFGF-23 levels had a higher 90-day mortality during the follow-up period with an adjusted HR of 1.76 (95% CI, 1.22–2.53; *p* = 0.020) as compared with patients with lower cFGF-23 values (Figure 3A). There was no interaction of the baseline comorbidities with high cFGF-23 to predict 90-day composite outcome. (Table S2) Taking mortality as a competing risk factor for dialysis, high cFGF-23 levels also predicted less weaning from dialysis in surviving patients (HR, 0.62, *p* = 0.032) (Figure 3B).

The relationship of cFGF-23 with these variables was also underscored by a GAM analysis adjusted for SOFA score, gender, and age, which showed that cFGF-23 levels correlated with iFGF-23 (*p* = 0.013) and SOFA score (*p* < 0.001), but not with sCr (*p* = 0.116), phosphate (*p* = 0.591), 25-hydroxy Vit D (*p* = 0.485) and 1, 25 dihydroxy vit D (*p* = 0.638) concentrations at initiating RRT (Figure S1).

### 3.5. Addition of cFGF-23 to AKI Risk Predicting Score at Start of Dialysis

Adding cFGF-23 to the AKI risk predicting score at dialysis initiation significantly increased risk stratification (total NRI = 0.148; 95% CI = 0.057–0.239; *p* = 0.002) for detection of 90-day mortality. This effect was primarily determined by death (NRI event = 0.068, 95% CI = 0.043–0.087; *p* = 0.025) and survival (NRI event = 0.069; 95% CI = 0.039–0.097; *p* = 0.029). Similarly, the total IDI was significant. (0.051, 95% CI = 0.024–0.079; *p <* 0.001) (Figures S2 and S3).

## 4. Discussion

At initializing dialysis, the discriminative power of AKI biomarkers for 90-day mortality is fair. At dialysis initiation, the discrimination of cFGF-23 is better than NGAL, KIM-1, iFGF-23 and creatinine predicting patients’ outcome. With mortality as competing risk, higher cFGF-23 levels also predicted lesser kidney recovery in survivors. More importantly, cFGF-23 had better predictive power than creatinine-adjusted urine NGAL and its integration into the AKI risk predicting score significantly enhanced the accuracy of risk stratification. At a cut-off level above 2050 RU/mL, cFGF-23 could predict of AKI mortality after adjusting for different clinical and disease severity parameters. Thus, cFGF-23 could be used as an early determinant of prognosis in ICU patients subjected at initializing RRT and also as an early determinant of the timing of dialysis initiation.

An increasing body of evidence has shown that cFGF-23 levels are increased in patients with AKI [11,14,29,30,31,32]. No significant interaction was observed between cFGF-23 and baseline CKD, sepsis grading in predicting mortality. The SOFA score was independently associated with increased cFGF-23 levels, which underpins the potential use of cFGF-23 in a critical care setting. We dare suggest that a higher plasma cFGF-23 not only corresponds with more severe AKI, but also reflects a higher degree of systemic inflammation.

Several mechanisms may explain increased FGF-23 levels in AKI: (1) increased production by osteocytes and possibly osteoblasts, that escapes regulation by parathyroid hormone, vitamin D signaling, and dietary phosphate restriction [33,34]; (2) increased ectopic production of FGF-23 by damaged renal tubules [33,35]; (3) tubular dysfunction resulting in FGF-23 resistance [36]; (4) and decreased clearance of circulating FGF-23 [14]. Whilst circulating FGF-23 levels rise rapidly during AKI [14] and a causal role for FGF-23 in the pathogenesis of left ventricular hypertrophy has previously been unveiled, suggesting that chronically elevated FGF-23 levels contribute directly to cardiac mortality in patients with CKD [37].

The ideal circumstances for whether and when to start RRT remain unclear [4]. We found significantly elevated cFGF-23 levels at the start of dialysis in non-survivors, whilst other structural and functional renal biomarkers failed to discriminate. Elevated plasma cFGF-23 was related to the degree of organ failure at initializing RRT [33]. In fact, high cFGF-23 concentrations predicted worse outcome equally well as the SOFA score in critically ill patients with advanced AKI [38]. Moreover, in patients without AKI, plasma cFGF-23 levels were significantly higher in the more severely ill patients [14]. This underscores that high cFGF-23 levels are correlated with increased systemic inflammation and/or stress secondary to illness or major surgery [33]. Although both serum and urine cFGF-23 could predict AKI mortality after ICU admission [12], many patients were oliguric at initializing dialysis, that will highlight the role of serum cFGF-23. In surviving patients, high cFGF-23 levels also predicted a lesser possibility for RRT withdrawal. Early prediction of renal recovery is likely to be helpful with regard to post-discharge care after critical illness and subsequent progression to CKD and ESRD.

Taken together, the ability of cFGF-23 to predict adverse outcomes might be related more to the systemic inflammatory status than to tubular damage. Based on our findings, a prognostic model can be constructed that allows to predict individual mortality risk as well as potential kidney recovery in surviving patients before starting RRT. The addition of cFGF-23 to a clinical AKI risk predicting score resulted in greater discrimination, and enhanced the ability to anticipate a higher number of subsequent deaths. Given the lack of appropriate or reliable biomarkers in patients receiving RRT, plasma cFGF-23 tentatively may serve as a novel outcome-specific marker in critical care nephrology. In patients with augmented plasma cFGF-23 concentration to arrive 2050 RU/mL, the clinician should evaluate the traditional AKI risk score or parameters to decide commencing dialysis.

Whether the cFGF-23 assay provides comparable sensitivity to that for iFGF-23 in patients with different stages of AKI or illness severity is still debated [13]. Although measurements obtained with iFGF-23 and cFGF-23 assays reflect the same circulating moiety, it has been suggested that the levels of iFGF23 also increased in patients who developed severe AKI, but the magnitude was lower than cFGF23 [13]. This is also supported by the present study showing that a plasma cFGF-23 concentration exceeding 2050 RU/mL at initializing RRT was significantly associated with worse patient outcome at a higher discriminative power than iFGF-23. The levels of adjusted urine cFGF23 also increased in patients who did not survive, but the magnitude was lower than serum cFGF23.

Several limitations of our study must be highlighted. Our cFGF-23 cutoff value was somewhat higher than that in other AKI studies [11,12,13,14,15], probably because most patients already had advanced AKI when admitted to the ICU. Furthermore, the predicting power of cFGF-23 in patients without AKI but with high inflammation status needs further validation. Finally, the exact mechanism underlying increased cFGF-23 concentrations in AKI patients as well as possible other intrinsic biological effects of cFGF-23 in this particular population remain to be explored. As previously studies few biomarkers were ever validated and they could only modestly predictive of renal recovery [8]; we do acknowledge also that the AUCs of cFGF-23 were relatively modest in AKI-D patients with critical status, however adding cFGF-23 to a parsimonious model augmented prediction of mortality and kidney recovery.

## 5. Conclusions

At initializing dialysis, the discriminative power of AKI biomarkers for 90-day mortality is fair. Our study showed that cFGF-23, measured at initiation of RRT in critical patients with AKI, may be a novel and distinct marker for predicting 90-day mortality after discharge and less weaning from RRT in survivors. Its predictive discrimination was superior to other established biomarkers of kidney injury, in particular creatinine, NGAL and Kim-1. Adding cFGF-23 to the traditional AKI risk predicting score may allow better risk stratification and enhance prognostic power. cFGF-23 could further be used as a surrogate marker to decide the best timing to initiate RRT.

## Figures and Tables

**Table 1:** Clinical characteristics of patient grouped by 90 days outcome.

	All	90-Day Survival	90-Day Mortality	*p*	90-Day Composite Outcome (−)	90-Day Composite Outcome (+)	*p*
	(*n* = 257)	(*n* = 122)	(*n* = 135)		(*n* = 76)	(*n* = 181)	
Patient characteristics							
Age	65.7 ± 16.6	63.4 ± 16.0	67.8 ± 16.9	0.035	61.3 ± 17.5	67.6 ± 15.9	0.005
Gender (male (%))	167 (65.0%)	82 (67.2%)	85 (63.0%)	0.514	54 (71.1%)	113 (62.4%)	0.200
Baseline creatinine (mg/dL)	2.0 ± 1.6	2.5 ± 1.9	1.5 ± 1.1	<0.001	1.8 ± 1.3	2.1 ± 1.7	0.220
eGFR (MDRD) (mL/min/1.73 m^2^)	55.6 ± 41.0	48.3 ± 44.2	62.2 ± 36.9	0.006	63.3 ± 47.6	52.3 ± 37.6	0.428
Co-morbidities							
Diabetes mellitus	115 (44.7%)	61 (50.0%)	54 (40.0%)	0.132	33 (43.4%)	82 (45.3%)	0.891
Cirrhosis	9 (3.5%)	3 (2.5%)	6 (4.4%)	0.505	2 (2.6%)	7 (3.9%)	0.999
COPD	15 (5.8%)	5 (4.1%)	10 (7.4%)	0.297	5 (6.6%)	10 (5.5%)	0.777
CAD	54 (21.0%)	24 (19.7%)	30 (22.2%)	0.648	18 (23.7%)	36 (19.9%)	0.505
CVA	24 (9.3%)	9 (7.4%)	15 (11.1%)	0.392	4 (5.3%)	20 (11.0%)	0.166
Congestive heart failure				0.683			0.780
0	67 (26.1%)	33 (27.0%)	34 (25.2%)		19 (25.0%)	48 (26.5%)	
I	100 (38.9%)	43 (35.2%)	57 (42.2%)		28 (36.8%)	72 (39.8%)	
II	51 (19.8%)	24 (19.7%)	27 (20.0%)		14 (18.4%)	37 (20.4%)	
III	31 (12.1%)	17 (13.9%)	14 (10.4%)		12 (15.8%)	19 (10.5%)	
Laboratory data at ICU admission							
BUN (mg/dL)	48.0 ± 33.5	58.1 ± 34.5	38.9 ± 29.9	<0.001	48.9 ± 36.6	47.7 ± 32.2	0.783
pH	7.4 ± 0.1	7.4 ± 0.8	7.4 ± 0.1	0.659	7.4 ± 0.1	7.4 ± 0.1	0.612
FiO_2_	0.5 ± 0.2	0.5 ± 0.2	0.5 ± 0.2	0.916	5 ± 0.2	0.5 ± 0.2	0.218
SBP (mmHg)	121.0 ± 28.4	129.8 ± 28.8	113.0 ± 25.6	<0.001	126.0 ± 25.6	118.8 ± 29.3	0.063
GCS	11.9 ± 4.2	12.3 ± 4.0	11.6 ± 4.4	0.164	11.9 ± 4.1	11.9 ± 4.3	0.948
SOFA	8.9 ± 3.5	8.3 ± 3.1	9.5 ± 3.7	0.008	8.7 ± 3.4	9.1 ± 3.6	0.410
APACHE II	16.3 ± 6.2	15.6 ± 6.0	9.5 ± 3.8	0.094	15.0 ± 6.4	16.9 ± 6.0	0.025
MODS	5.9 ± 3.7	5.5 ± 3.4	6.4 ± 3.8	0.040	5.7 ± 3.3	6.0 ± 3.8	0.507
Etiology of AKI							
Shock	150 (58.4%)	56 (5.9%)	94 (69.6)	<0.001	40 (52.6%)	110 (60.8%)	0.268
Sepsis	98 (38.1%)	26 (23.8%)	69 (51.1%)	<0.001	22 (28.9%)	76 (42.0%)	0.067
Drug-induced	3 (1.2%)	0 (0%)	3 (2.2%)	0.249	0 (0%)	3 (1.7%)	0.557
Rhabdomyolysis	9 (3.5%)	5 (4.1%)	4 (3.0%)	0.740	4 (5.3%)	5 (2.8%)	0.457
Pigmentation	6 (2.3%)	4 (3.3%)	2 (1.5%)	0.427	4 (5.3%)	2 (1.1%)	0.065
Contrast	37 (14.4%)	22 (18.0%)	15 (11.1%)	0.154	13 (17.1%)	24 (13.3%)	0.440
Other	26 (10.1%)	16 (13.1%)	10 (7.4%)	0.150	7 (9.2%)	19 (10.5%)	0.825
At initiating dialysis							
Admission to dialysis (days)	40.3 ± 27.1	42.0 ± 31.8	37.1 ± 47.5	0.335	45.8 ± 33.9	36.8 ± 43.1	0.106
Mechanical Ventilation	185 (72.0%)	74 (60.7%)	111 (82.2%)	<0.001	49 (64.5%)	136 (75.1%)	0.095
Emergency Surgery	100 (38.9%)	49 (40.2%)	51 (37.8%)	0.703	33 (43.4%)	67 (37.0%)	0.400
IABP	27 (10.5%)	10 (8.2%)	17 (12.6%)	0.310	7 (9.2%)	20 (11.0%)	0.824
Urine output (mL/24 h)	591.7 ± 790.3	750.3 ± 1013.0	448.3 ± 472.1	0.002	869.7 ± 1188.7	474.9 ± 503.1	<0.001
AKI risk prediction score	22.6 ± 6.9	20.2 ± 6.5	24.9 ± 6.5	<0.001	20.8 ± 6.4	23.4 ± 7.0	0.004
Body weight (kg)	66.8 ± 14.3	68.6 ± 15.9	67.8 ± 16.9	0.055	70.0 ± 15.9	65.5 ± 13.4	0.021
IE	8.2 ± 15.0	4.7 ± 8.3	11.3 ± 18.7	<0.001	5.24 ± 9.32	9.43 ± 16.75	0.041
SOFA	10.9 ± 3.9	9.1 ± 3.2	12.6 ± 3.8	<0.001	9.4 ± 3.3	11.6 ± 4.0	<0.001
APACHE II	17.8 ± 6.4	15.6 ± 5.4	19.8 ± 6.7	<0.001	15.5 ± 5.7	18.7 ± 6.5	<0.001
MODS	8.1 ± 4.1	6.5 ± 3.7	9.5 ± 3.9	<0.001	7.0 ± 3.6	8.6 ± 4.2	0.005
Phosphate (mg/dL)	4.5 ± 1.7	4.8 ± 1.6	4.3 ± 1.8	0.085	4.8 ± 1.5	4.4 ± 1.8	0.333
25 OH Vit D, ng/mL	11.7 ± 5.6	10.8 ± 5.5	12.9 ± 5.9	0.471	11.2 ± 7.1	12.0 ± 5.2	0.812
1,25 diOH Vit D, pg/mL	27.3 ± 6.5	25.5 ± 6.4	29.7 ± 6.4	0.545	28.9 ± 6.8	26.6 ± 6.6	0.545
Kidney function marker							
BUN (mg/dL)	82.4 ± 47.2	82.7 ± 51.5	82.5 ± 45.4	0.922	82.3 ± 51.5	82.5 ± 45.4	0.978
Creatinine (mg/dL)	2.0 ± 1.6	4.1 ± 2.2	4.2 ± 2.4	0.745	4.1 ± 2.2	4.2 ± 2.4	0.745
Urine NGAL (ng/mL)	197.5 ± 85.3	191.0 ± 93.3	203.5 ± 77.1	0.254	189.2 ± 97.6	201.0 ± 79.7	0.330
Urine NGAL/Cre	6.9 ± 11.1	6.8 ± 12.5	6.9 ± 9.7	0.912	5.0 ± 6.9	7.7 ± 12.4	0.085
Urine KIM1 (ng/mL)	6.0 ± 5.8	5.8 ± 5.8	6.2 ± 5.8	0.529	5.9 ± 6.5	5.7 ± 5.4	0.139
Urine KIM1/Cre	0.1 ± 0.2	0.1 ± 0.2	0.1 ± 0.1	0.993	0.1 ± 0.1	0.1 ± 0.2	0.699
Urine cFGF-23/Cre	877.4 ± 994.3	671.4 ± 924.9	1063.5 ± 1021.2	<0.001	699.1 ± 1015.0	952.2 ± 978.6	0.062
Plasma iFGF-23 (pg/mL)	304.2 ± 468.0	395.1 ± 635.6	269.0 ± 385.2	0.265	320.4 ± 551.8	300.2 ± 449.5	0.875
Plasma cFGF-23 (RU/mL)	2630.1 ± 2259.5	1926.7 ± 1745.4	3265.9 ± 2479.0	<0.001	1925.3 ± 1917.3	2926.1 ± 2330.0	0.001
Indication for dialysis							
Azotemia	123 (47.9%)	58 (47.5%)	65 (48.1%)	0.999	32 (42.1%)	91 (50.3%)	0.274
Fluid overload	111 (43.2%)	51 (41.8%)	60 (44.4%)	0.706	30 (39.5%)	81 (44.8%)	0.491
Electrolyte disorders	18 (7.0%)	10 (8.2%)	18 (5.9%)	0.626	7 (9.2%)	11 (6.1%)	0.423
Metabolic acidosis	46 (17.9%)	17 (13.9%)	29 (21.5%)	0.143	11 (14.5%)	35 (19.3%)	0.380
Oliguria	166 (64.6%)	69 (56.6%)	97 (71.9%)	0.013	46 (56.6%)	123 (68.0%)	0.088
Uremic encephalopathy	12 (4.7%)	9 (7.4%)	3 (2.2%)	0.074	6 (7.9%)	6 (3.3%)	0.191
Dialysis modality							
CVVH	62 (21.1%)	16 (13.1)	46 (34.1%)	<0.001	15 (19.7%)	47 (26.0%)	0.296
IHD	62 (29.2%)	47 (38.5%)	28 (20.7%)		27 (35.5%)	48 (26.5%)	
SLED	120 (46.7%)	59 (48.5%)	61 (45.2%)		34 (44.7%)	86 (47.5%)	
Relevant outcome parameters							
Hospital length of stay (days)	54.7 ± 50.4	52.3 ± 41.1	56.9 ± 57.6	0.459	59.0 ± 46.3	52.9 ± 52.0	0.383
Duration of hospital dialysis (days)	82.4 ± 60.7	42.0 ± 31.8	37.1 ± 47.5	0.335	45.8 ± 33.9	36.8 ± 43.1	0.745

**Abbreviations**: AKI, acute kidney injury; APACHE; Acute Physiology and Chronic Health Evaluation, BMI, body mass index; CABG, coronary artery bypass graft; BUN, blood urea nitrogen; COPD, chronic obstructive pulmonary disease; CPB, cardiopulmonary bypass; Cre, creatinine; CVA, cerebrovascular accident; CVVH, continuous venovenous hemofiltration; eGFR, estimated glomerular filtration rate; FGF-23, Fibroblast growth factor-23; GCS, Glasgow Coma Scale; IABP: intra-aortic balloon pump; IE, inotropic equivalent; ICU, intensive care unit; IHD, intermittent hemodialysis; KIM-1, Kidney Injury Molecule-1; LVEF, Left ventricular ejection fraction; MDRD, Modification of Diet in Renal Disease; MODS, Multiple Organ Dysfunction Syndrome; NGAL, neutrophil gelatinase-associated lipocalin; SLED, sustained low efficiency dialysis; SOFA, Sequential Organ Failure Assessment; Vit D, vitamin D.

**Table 2:** Clinical characteristics of patients with high versus low plasma cFGF-23 levels.

Serum cFGF23 Categories	Low cFGF-23	High cFGF-23	*p*
	(*n* = 116)	(*n* = 141)	
Patient characteristics			
Age (years)	65.8 ± 16.0	65.7 ± 17.2	0.973
Gender (male)	77 (66.4%)	90 (63.8%)	0.695
Baseline creatinine (mg/dL)	2.2 ± 1.9	1.8 ± 1.3	0.039
eGFR (MDRD) (mL/min/1.73 m^2^)	54.4 ± 42.5	56.5 ± 39.8	0.690
Comorbidities			
Diabetes mellitus	33 (43.4%)	82 (45.3%)	0.891
Cirrhosis	1 (0.9%)	8 (5.7%)	0.044
COPD	7 (6.0%)	8 (5.7%)	0.999
CAD	27 (23.3%)	27 (19.1%)	0.445
CVA	12 (10.3%)	12 (8.5%)	0.670
Congestive heart failure			0.265
0	32 (27.6%)	35 (24.8%)	
I	37 (31.9%)	63 (44.7%)	
II	27 (23.3%)	24 (17.0%)	
III	15 (12.9%)	16 (11.3%)	
IV	0 (0%)	8 (5.5%)	
Laboratory data at ICU admission			
BUN (mg/dL)	48.3 ± 36.1	47.8 ± 31.4	0.897
pH	7.4 ± 0.1	7.4 ± 0.1	0.354
FiO2	0.5 ± 0.2	0.5 ± 0.2	0.609
SBP	126.1 ± 29.2	116.7 ± 27.0	0.008
GCS	11.9 ± 4.3	11.9 ± 4.2	0.984
SOFA	8.2 ± 3.6	9.6 ± 3.3	0.001
APACHE II	15.9 ± 6.0	16.6 ± 6.4	0.405
MODS	5.7 ± 3.5	6.1 ± 3.8	0.328
Etiology of AKI			
Shock	66 (56.9%)	84 (59.6%)	0.704
Sepsis	40 (34.5%)	58 (41.1%)	0.303
Rhabdomyolysis	7 (6.0%)	2 (1.4%)	0.083
Drug-induced	2 (1.7%)	1 (0.7%)	0.591
Pigmentation	5 (4.3%)	1 (0.7%)	0.094
Contrast	17 (14.7%)	20 (14.2%)	0.999
Others	12 (10.3%)	14 (9.9%)	0.999
At initiating dialysis			
Admission to dialysis (days)	35.5 ± 34.1	42.6 ± 45.4	0.163
Mechanical ventilation	78 (67.2%)	107 (75.9%)	0.128
Emergency Surgery	45 (38.8%)	55 (39.0%)	0.999
IABP	13 (11.2%)	14 (9.9%)	0.839
Urine output (mL/24 h)	650.9 ± 642.9	542.9 ± 892.8	0.277
AKI risk prediction score	21.5 ± 6.7	23.5 ± 6.9	0.021
Body weight (kg)	67.2 ± 15.4	66.5 ± 13.3	0.728
IE	7.14 ± 11.5	9.1 ± 17.4	0.310
SOFA	10.6 ± 4.3	11.2 ± 3.6	0.218
APACHE II	17.8 ± 6.7	11.8 ± 6.2	0.980
MODS	7.8 ± 4.4	8.4 ± 3.8	0.222
Phosphate, mg/dL	4.1 ± 1.7	4.9 ± 1.7	0.021
25 OH Vit D, ng/mL	11.0 ± 5.8	12.5 ± 5.6	0.617
1,25 diOH Vit D, pg/mL	29.7 ± 6.9	25.0 ± 5.5	0.149
Kidney function marker			
BUN (mg/dL)	81.2 ± 45.8	83.4 ± 48.4	0.714
Creatinine (mg/dL)	4.2 ± 2.4	4.1 ± 2.3	0.677
Urine KIM1 (ng/mL)	5.9 ± 5.9	6.1 ± 5.7	0.800
Urine KIM1/Cre	0.13 ± 0.18	0.14 ± 0.14	0.715
Urine NGAL (ng/mL)	196.5 ± 86.1	198.2 ± 85.0	0.916
Urine NGAL/Cre	7.0 12.9	6.8 ± 9.4	0.877
Urine cFGF-23/Cre	523.4 ± 747.2	1173.3 ± 1077.6	<0.001
Plasma iFGF-23 (pg/mL)	257.6 ± 243.0	325.50 ± 542.3	0.536
Indication for dialysis			
Azotemia	56 (48.3%)	67 (47.5%)	0.999
Fluid overload	48 (41.4%)	63 (44.7%)	0.615
Electrolyte disorders	7 (60%)	11 (7.8%)	0.631
Metabolic acidosis	22 (19.0%)	24 (17.0%)	0.745
Oliguria	73 (62.9%)	93 (66.0%)	0.694
Uremic complication	7 (6.0%)	5 (3.5%)	0.386
Dialysis modality			0.011
CVVH	44 (37.9%)	31 (22.0%)	
IHD	21 (18.1%)	41 (29.1%)	
SLED	51 (44.0%)	69 (48.9%)	
Outcomes of interest			
Hospital length of stay (days)	49.0 ± 43.0	59.4 ± 55.5	0.101
Duration of hospital dialysis (days)	39.9 ± 34.4	39.0 ± 45.5	0.862
Hospital mortality	42 (36.2%)	82 (58.2%)	<0.001
Composite outcome at discharge	69 (59.5%)	104 (73.8%)	<0.001
90-day mortality	45 (38.8%)	90 (63.8%)	<0.001
90-day weaning from dialysis	47 (40.5%)	29 (20.6%)	<0.001
90-day composite outcome	69 (59.5%)	112 (79.4%)	<0.001

**Abbreviations:** AKI, acute kidney injury; APACHE; Acute Physiology and Chronic Health Evaluation, BMI, body mass index; CABG, coronary artery bypass graft; Cre, creatinine; BUN, blood urea nitrogen; COPD, chronic obstructive pulmonary disease; CPB, cardiopulmonary bypass; Cre, creatinine; CVA, cerebrovascular accident; CVVH, continuous venovenous hemofiltration; eGFR, estimated glomerular filtration rate; FGF-23, Fibroblast growth factor-23; GCS, Glasgow Coma Scale; IABP: intra-aortic balloon pump; IE, inotropic equivalent; ICU, intensive care unit; IHD, intermittent hemodialysis; KIM-1, Kidney Injury Molecule-1; LVEF, Left ventricular ejection fraction; MDRD, Modification of Diet in Renal Disease; MODS, Multiple Organ Dysfunction Syndrome; NGAL, neutrophil gelatinase-associated lipocalin; SLED, sustained low efficiency dialysis; SOFA, Sequential Organ Failure Assessment; Vit D, vitamin D.

**Table 3:** Logistic regression model for mortality and composite outcomes at hospital discharge and 90 days after discharge. Significant risks were shown.

Independent Variables	Hospital Mortality			Composite Outcome at Discharge		
	OR	95% CI	*p*	OR	95% CI	*p*
Age (per year)	1.03	1.01–1.04	0.007	1.03	1.01–1.04	0.004
SOFA (per score)	1.26	1.15–1.39	<0.001	1.12	1.03–1.22	0.011
High cFGF-23	1.80	1.01–3.24	0.043	1.80	1.01–3.19	0.045
	**90-Day Mortality**			**90-Day Composite Outcome**		
Age (per year)	1.03	1.01–1.05	0.001	1.03	1.01–1.05	0.001
SOFA (per score)	1.30	1.17–1.44	0.037	1.17	1.07–1.27	<0.001
High cFGF-23	2.19	1.20–4.00	0.011	2.39	1.31–4.35	0.005

**Abbreviations**: cFGF-23, c-terminal fibroblast growth factor-23; CI, confidence interval; HR, hazard ratio; SOFA, Sequential Organ Failure Assessment. All the univariate significant and non-significant relevant covariates, including age, sex, baseline comorbidities, indication for dialysis, etiology of AKI, kidney function profile (e.g., baseline eGFR and candidate biomarkers), cFGF-23 and SOFA score at dialysis initiation, dialysis modality, and some of their interactions were put on the variable lists to be selected (Table 1).

## Supplementary Materials

The following are available online at http://www.mdpi.com/2077-0383/7/8/202/s1, Figure S1: Scatter plots with an adjusted spline of cFGF23 with (A) iFGF23 (*p* = 0.013), (B) phosphate (*p* = 0.591) (C) creatinine (*p* = 0.116) (D) 25 OH Vitamin D (*p* = 0.485) and (E) 1,25 OH, Vitamin D (*p* = 0.638) (F) KDIGO-AKI score (*p* = 0.820) (G) SOFA (*p* < 0.001) at initiation of dialysis, Figure S2: Decision curve analysis (DCA) plot to assess 90 day mortality using cFGF-23 in addition to AKI risk prediction score, Figure S3: The correlation of AKI risk predicting score and AKI risk predicting score with cFGF-23 predicting 90 day mortality, Table S1: *p* value comparison of the receiver operating characteristic (ROC) curve for discriminative ability, Table S2: Interaction of baseline co-morbidity with high cFGF-23 to predict 90-day composite outcome.

## Abbreviations

AKI	acute kidney injury
APACHE II	Acute Physiology and Chronic Health Evaluation II
AUROC	area under the receiver operator characteristic curve
CKD	chronic kidney disease
ESRD	end-stage renal disease
ICU	intensive care unit
KIM-1	Kidney Injury Molecule-1
MODS	Multiple Organ Dysfunction Score
NGAL	neutrophil gelatinase-associated lipocalin
RRT	renal replacement therapy
RU	relative units
sCr	serum creatinine
SOFA	Sequential Organ Failure Assessment

## References

[1] Shiao C.C., Wu P.C., Huang T.M., Lai T.S., Yang W.S., Wu C.H., Lai C.F., Wu V.C., Chu T.S., Wu K.D. (2015). Long-term remote organ consequences following acute kidney injury. Crit. Care.

[2] Wu V.C., Shiao C.C., Chang C.H., Huang T.M., Lai C.F., Lin M.C., Chiang W.C., Chu T.S., Wu K.D., Ko W.J. (2014). Long-term outcomes after dialysis-requiring acute kidney injury. Biomed. Res. Int..

[3] Wald R., Bagshaw S.M. (2014). The timing of renal replacement therapy initiation in acute kidney injury: Is earlier truly better?. Crit. Care Med..

[4] Klein S.J., Brandtner A.K., Lehner G.F., Ulmer H., Bagshaw S.M., Wiedermann C.J., Joannidis M. (2018). Biomarkers for prediction of renal replacement therapy in acute kidney injury: A systematic review and meta-analysis. Intensive Care Med..

[5] Kumpers P., Hafer C., Lukasz A., Lichtinghagen R., Brand K., Fliser D., Faulhaber-Walter R., Kielstein J.T. (2010). Serum neutrophil gelatinase-associated lipocalin at inception of renal replacement therapy predicts survival in critically ill patients with acute kidney injury. Crit. Care.

[6] Zarbock A., Kellum J.A., Schmidt C., Van Aken H., Wempe C., Pavenstadt H., Boanta A., Gerss J., Meersch M. (2016). Effect of Early vs Delayed Initiation of Renal Replacement Therapy on Mortality in Critically Ill Patients With Acute Kidney Injury: The Elain Randomized Clinical Trial. JAMA.

[7] Lin C.Y., Chang C.H., Fan P.C., Tian Y.C., Chang M.Y., Jenq C.C., Hung C.C., Fang J.T., Yang C.W., Chen Y.C. (2013). Serum interleukin-18 at commencement of renal replacement therapy predicts short-term prognosis in critically ill patients with acute kidney injury. PLoS ONE.

[8] Pike F., Murugan R., Keener C., Palevsky P.M., Vijayan A., Unruh M., Finkel K., Wen X., Kellum J.A. (2015). Biomarker Enhanced Risk Prediction for Adverse Outcomes in Critically Ill Patients Receiving RRT. Clin. J. Am. Soc. Nephrol..

[9] Consortium A. (2000). Autosomal dominant hypophosphataemic rickets is associated with mutations in FGF23. Nat. Genet..

[10] Berndt T., Kumar R. (2007). Phosphatonins and the regulation of phosphate homeostasis. Annu. Rev. Physiol..

[11] Ali F.N., Hassinger A., Price H., Langman C.B. (2013). Preoperative plasma FGF23 levels predict acute kidney injury in children: Results of a pilot study. Pediatr. Nephrol..

[12] Leaf D.E., Jacob K.A., Srivastava A., Chen M.E., Christov M., Juppner H., Sabbisetti V.S., Martin A., Wolf M., Waikar S.S. (2017). Fibroblast Growth Factor 23 Levels Associate with AKI and Death in Critical Illness. J. Am. Soc. Nephrol..

[13] Leaf D.E., Christov M., Juppner H., Siew E., Ikizler T.A., Bian A., Chen G., Sabbisetti V.S., Bonventre J.V., Cai X. (2016). Fibroblast growth factor 23 levels are elevated and associated with severe acute kidney injury and death following cardiac surgery. Kidney Int..

[14] Christov M., Waikar S.S., Pereira R.C., Havasi A., Leaf D.E., Goltzman D., Pajevic P.D., Wolf M., Juppner H. (2013). Plasma FGF23 levels increase rapidly after acute kidney injury. Kidney Int..

[15] Donate-Correa J., de Fuentes M.M., Mora-Fernandez C., Navarro-Gonzalez J.F. (2014). Pathophysiological implications of fibroblast growth factor-23 and Klotho and their potential role as clinical biomarkers. Clin. Chem..

[16] Wu V.C., Ko W.J., Chang H.W., Chen Y.S., Chen Y.W., Chen Y.M., Hu F.C., Lin Y.H., Tsai P.R., Wu K.D. (2007). Early renal replacement therapy in patients with postoperative acute liver failure associated with acute renal failure: Effect on postoperative outcomes. J. Am. Coll. Surg..

[17] Wu V.C., Ko W.J., Chang H.W., Chen Y.W., Lin Y.F., Shiao C.C., Chen Y.M., Chen Y.S., Tsai P.R., Hu F.C. (2008). Risk factors of early redialysis after weaning from postoperative acute renal replacement therapy. Intensive Care Med..

[18] Shiao C.C., Wu V.C., Li W.Y., Lin Y.F., Hu F.C., Young G.H., Kuo C.C., Kao T.W., Huang D.M., Chen Y.M. (2009). Late initiation of renal replacement therapy is associated with worse outcomes in acute kidney injury after major abdominal surgery. Crit. Care.

[19] Wu V.C., Wang C.H., Wang W.J., Lin Y.F., Hu F.C., Chen Y.W., Chen Y.S., Wu M.S., Lin Y.H., Kuo C.C. (2010). Sustained low-efficiency dialysis versus continuous veno-venous hemofiltration for postsurgical acute renal failure. Am. J. Surg..

[20] Huang T.M., Wu V.C., Young G.H., Lin Y.F., Shiao C.C., Wu P.C., Li W.Y., Yu H.Y., Hu F.C., Lin J.W. (2011). Preoperative proteinuria predicts adverse renal outcomes after coronary artery bypass grafting. J. Am. Soc. Nephrol..

[21] Demirjian S., Chertow G.M., Zhang J.H., O’Connor T.Z., Vitale J., Paganini E.P., Palevsky P.M. (2011). Network VNARFT. Model to predict mortality in critically ill adults with acute kidney injury. Clin. J. Am. Soc. Nephrol..

[22] Wu V.C., Huang T.M., Lai C.F., Shiao C.C., Lin Y.F., Chu T.S., Wu P.C., Chao C.T., Wang J.Y., Kao T.W. (2011). Acute-on-chronic kidney injury at hospital discharge is associated with long-term dialysis and mortality. Kidney Int..

[23] Lin Y.F., Ko W.J., Wu V.C., Chen Y.S., Chen Y.M., Hu F.C., Shiao C.C., Wu M.S., Chen Y.W., Li W.Y. (2008). A modified sequential organ failure assessment score to predict hospital mortality of postoperative acute renal failure patients requiring renal replacement therapy. Blood Purif..

[24] Shiao C.C., Ko W.J., Wu V.C., Huang T.M., Lai C.F., Lin Y.F., Chao C.T., Chu T.S., Tsai H.B., Wu P.C. (2012). U-curve association between timing of renal replacement therapy initiation and in-hospital mortality in postoperative acute kidney injury. PLoS ONE.

[25] Wu V.C., Lo S.C., Chen Y.L., Huang P.H., Tsai C.T., Liang C.J., Kuo C.C., Kuo Y.S., Lee B.C., Wu E.L. (2011). Endothelial progenitor cells in primary aldosteronism: A biomarker of severity for aldosterone vasculopathy and prognosis. J. Clin. Endocrinol. Metab..

[26] Wu V.C., Lai C.F., Shiao C.C., Lin Y.F., Wu P.C., Chao C.T., Hu F.C., Huang T.M., Yeh Y.C., Tsai I.J. (2012). Effect of diuretic use on 30-day postdialysis mortality in critically ill patients receiving acute dialysis. PLoS ONE.

[27] Hin L.Y., Lau T.K., Rogers M.S., Chang A.M. (1999). Dichotomization of continuous measurements using generalized additive modelling—Application in predicting intrapartum caesarean delivery. Stat. Med..

[28] Wu V.C., Chang C.H., Wang C.Y., Lin Y.H., Kao T.W., Lin P.C., Chu T.S., Chang Y.S., Chen L., Wu K.D. (2017). Risk of Fracture in Primary Aldosteronism: A Population-Based Cohort Study. J. Bone Miner. Res..

[29] Zhang M., Hsu R., Hsu C.Y., Kordesch K., Nicasio E., Cortez A., McAlpine I., Brady S., Zhuo H., Kangelaris K.N. (2011). FGF-23 and PTH levels in patients with acute kidney injury: A cross-sectional case series study. Ann. Intensive Care..

[30] Leaf D.E., Wolf M., Waikar S.S., Chase H., Christov M., Cremers S., Stern L. (2012). FGF-23 levels in patients with AKI and risk of adverse outcomes. Clin. J. Am. Soc. Nephrol..

[31] Leaf D.E., Waikar S.S., Wolf M., Cremers S., Bhan I., Stern L. (2013). Dysregulated mineral metabolism in patients with acute kidney injury and risk of adverse outcomes. Clin. Endocrinol..

[32] Brown J.R., Katz R., Ix J.H., de Boer I.H., Siscovick D.S., Grams M.E., Shlipak M., Sarnak M.J. (2014). Fibroblast growth factor-23 and the long-term risk of hospital-associated AKI among community-dwelling older individuals. Clin. J. Am. Soc. Nephrol..

[33] Neyra J.A., Moe O.W., Hu M.C. (2015). Fibroblast growth factor 23 and acute kidney injury. Pediatr. Nephrol..

[34] Shimada T., Muto T., Urakawa I., Yoneya T., Yamazaki Y., Okawa K., Takeuchi Y., Fujita T., Fukumoto S., Yamashita T. (2002). Mutant FGF-23 responsible for autosomal dominant hypophosphatemic rickets is resistant to proteolytic cleavage and causes hypophosphatemia in vivo. Endocrinology.

[35] Spichtig D., Zhang H., Mohebbi N., Pavik I., Petzold K., Stange G., Saleh L., Edenhofer I., Segerer S., Biber J. (2014). Renal expression of FGF23 and peripheral resistance to elevated FGF23 in rodent models of polycystic kidney disease. Kidney Int..

[36] Hassan A., Durlacher K., Silver J., Naveh-Many T., Levi R. (2016). The fibroblast growth factor receptor mediates the increased FGF23 expression in acute and chronic uremia. Am. J. Physiol. Renal Physiol..

[37] Faul C., Amaral A.P., Oskouei B., Hu M.C., Sloan A., Isakova T., Gutierrez O.M., Aguillon-Prada R., Lincoln J., Hare J.M. (2011). FGF23 induces left ventricular hypertrophy. J. Clin. Investig..

[38] Chang C.H., Fan P.C., Chang M.Y., Tian Y.C., Hung C.C., Fang J.T., Yang C.W., Chen Y.C. (2014). Acute kidney injury enhances outcome prediction ability of sequential organ failure assessment score in critically ill patients. PLoS ONE.

