# Supplementary material for: Outcome Prediction of Acute Kidney Injury Biomarkers at Initiation of Dialysis in Critical Units
Source: J Clin Med. 2018 Aug 6;7(8):202. doi: 10.3390/jcm7080202 (PMC6112021; doi:10.3390/jcm7080202)
Supplement: Supplementary file 1 [file jcm-07-00202-s001.pdf]

Additional materials.

The data collection in this study was approved by the Institutional Research Ethics Review Boards (201409024RINB in National Taiwan University Hospital, 01-X16-059 in Buddhist Tzu Chi General Hospital, and TYGH104007 in Taoyuan General Hospital). Enrollment was performed in a consecutively randomized fashion after obtaining written informed consent from the patients or their legal representatives. All methods were carried out in accordance with approved guidelines.

Definition of organ failure

Definitions of organ failure were as follows: diabetes mellitus, previous use of insulin or oral hypoglycemic agents; hypertension, use of anti-hypertensive agents or blood pressure > 140/90 mmHg at the time of hospitalization; heart failure, New York Heart Association functional class IV; chronic kidney disease, baseline eGFR ≦ 60 $mL/min/1.73m^2$ for more than 3 months; sepsis, persisting or progressive signs and symptoms of the systemic inflammatory response syndrome with clinical evidence of infection (1).

Disease severity score

At initiation of RRT, Acute Physiology and Chronic Health Evaluation II (APACHE II) (2), Sequential Organ Failure Assessment (SOFA) (3) and Multiple Organ

Dysfunction (MODS) scores were calculated and the inotropic equivalent [dopamine + dobutamine + (epinephrine + norepinephrine + isoproterenol) x 100 + milrinone x15 (mcg/kg/min)] determined (4).

Indication for dialysis

The pre-determined indications for RRT were: (1) presence of azotemia [blood urea nitrogen (BUN) > 80 mg/dl and serum creatinine (sCr) > 2 mg/dl) with uremic symptoms (encephalopathy, pericarditis or pleurisy); (2) oliguria (urine output < 400 mL/24h) or anuria refractory to diuretics; (3) fluid overload refractory to diuretics with a central venous pressure (CVP) > 12 mmHg or pulmonary edema with a PaO_2/FiO_2 < 300 mmHg; (4) hyperkalemia (serum potassium >5.5 mmol/l) refractory to medical treatment, and/or (5) metabolic acidosis (aterial pH < 7.2).(5-7)

Dialysis methods

Patients who needed the inotropic equivalent (IE) of more than 15 mcg/kg/min to maintain systolic blood pressure above 120 mmHg, received continuous venovenous hemofiltration (CVVH). Hemofiltration flow and blood flows were 25mL/kg/h and 200 mL/min, respectively. Replacement fluid was bicarbonate-buffered and predilutionally administered at a dynamically adjusted rate to achieve the desired fluid therapy goals. In patients who required an IE of 5-15 mcg/kg/min, sustained low-efficiency daily dialysis (SLEDD) or diafiltration (SLEDD-f) was used with a blood flow of 200mL/min, a dialysate flow of 300mL/min, and a hemofiltration flow

of 25mL/kg/h. Duration of treatment was around 6-12 h depending on the amount of ultrafiltration. Intermittent hemodialysis was performed for four h (except for the first and second session) using low-flux polysulphone hemofilter (KF-18C, Kawasumi Laboratories, Japan), with a dialysate and blood flow of 500mL/min. (5, 7-9). RRT was performed via a double-lumen central venous catheter in all patients.

cFGF-23 and iFGF-23 measurement

The results yielded by the FGF-23 C-terminal kit were denoted as "cFGF-23" in the current study, which was the combination of the C-terminal fragments and intact FGF-23 (iFGF-23).(10)

Statistical methods

Continuous data were expressed as mean ± standard deviation (SD) and group comparisons were conducted using χ 2 tests for equal proportions, t tests for normally distributed data, and Wilcoxon rank sum tests otherwise. We generated receiver-operating characteristics (ROC) curves and calculated the area under the curve (AUC) to measure the performance of candidate criteria. Multiple comparisons were analyzed using one-way analysis of variance (ANOVA).

All the relevant covariates, including characteristics, comorbidities, laboratory data, at ICU admission, etiology of AKI, indication for dialysis, dialysis modality, SOFA

score, and plasma cFGF-23 at dialysis, and some of their interactions, such as interventions listed in table 1, were put on a selected variable list to predict the outcome of interest. To avoid the extremely over-fitted models, we did not put in outcome, GCS, APACHE II, MODS and kidney function markers other than urine cFGF-23. The significance levels for entry (SLE) and stay (SLS) were conservatively set at 0.15. Then, with the aid of substantive knowledge, the best candidate final logistic regression model was identified manually by dropping the covariates with $p > 0.05$ until all regression coefficients were significantly different from 0.

Survival curves for all-cause mortality or liberation from dialysis were plotted from adjusted Cox models. For long-term dialysis, an individual who survived at index discharge was censored at death or the end of the study period.

Assessing the performance of prediction models, decision curve analysis (DCA)

Clinical usefulness and net benefit of the cFGF-23 were estimated according with decision curve analyses (DCA)(11), in order to identify patients who will have any of the adverse events evaluated. The DCA show the clinical usefulness of each new model based on a continuum of potential thresholds for adverse events (x-axis) and the net benefit of using the model to stratify patients at risk (y-axis) relative to assuming that no patient will have an adverse event. The basic interpretation of DCA

is that the strategy with the highest net benefit at a particular threshold probability has the highest clinical value. In this study, the prediction models are represented by dot lines (AKI risk prediction score) and dashed lines (cFGF-23 and AKI risk prediction score). Those models that are the farthest away from the slanted horizontal grey line (i.e., assume none adverse event) and the slanted balck line (i.e., assume all adverse events) and the horizontal black line (i.e., assume none adverse event) demonstrate the higher net clinical benefit.

All analyses were performed with R software, version 3.2.2 (Free Software Foundation, Inc., Boston, MA), MedCalc Statistical Software, version 15.11.3 (MedCalc Software bvba, Ostend, Belgium; https://www.medcalc.org; 2015) and Stata/MP version 12 (Stata Corporation, TX) for competing-risk analysis. A p-value <0.05 was considered significant.

Study limitations

Unmeasured confounders which dodged thorough statistical risk factor adjustment could have influenced the results. Repeated cFGF-23 measurements instead of a single sampling at start of RRT might have provided more insight in evolution and possible changes of this biomarker over time. cFGF-23 may thus not only be useful

for early recognition of patients at high risk of AKI, but could also be applied as risk stratification factor to reduce diagnostic uncertainty.

Legends

Supplementary Figure 1. Scatter plots with an adjusted spline of cFGF23 with (A) iFGF23 (P= 0.013), (B) phosphate (p= 0.591) (C) creatinine (p=0.116) (D) 25 OH Vitamin D (p= 0.485) and (E)1, 25 OH, Vitamin D (p= 0.638) (F) KDIGO-AKI score (p=0.820) (G) SOFA (p<0.001) at initiation of dialysis.

The plots were incorporated with the subject-specific (longitudinal) random effects to predict the association constructed with the generalized additive model (GAM).

*Adjusted by sex, age, and SOFA score.

(A)

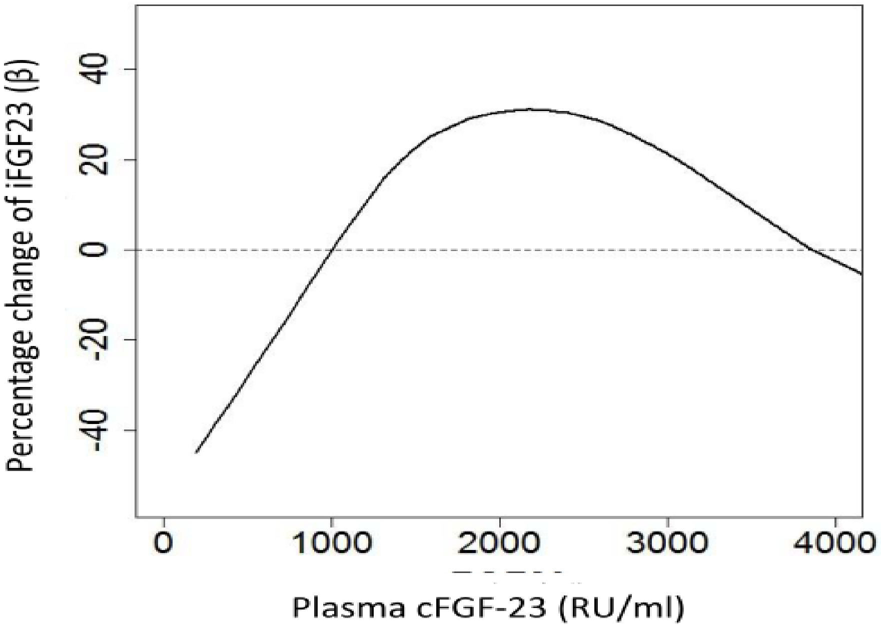

(B)

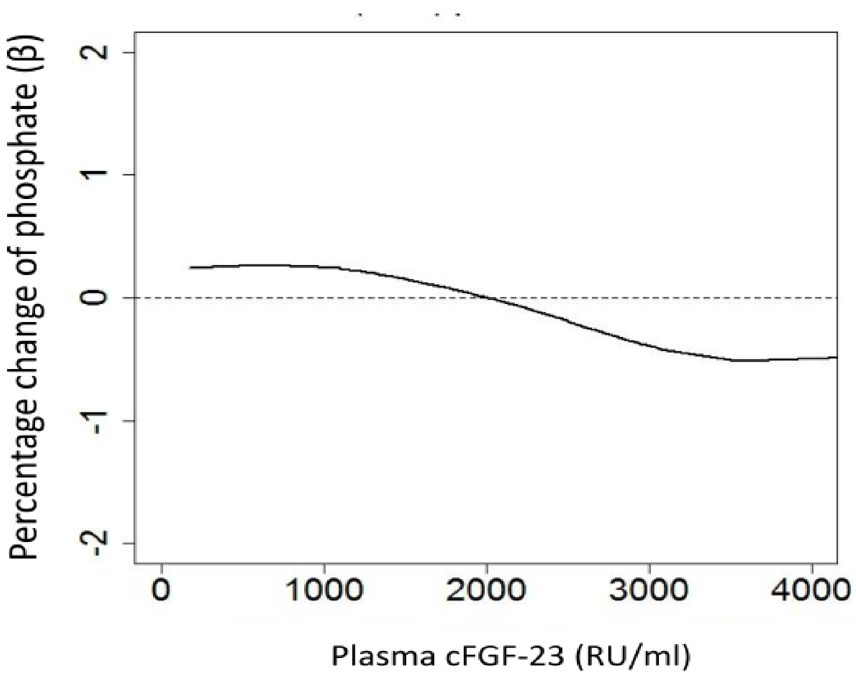
Percentage change of phosphate (β)
2
1
0
-1
-2
0
1000
2000
3000
4000
Plasma cFGF-23 (RU/ml)

(C)

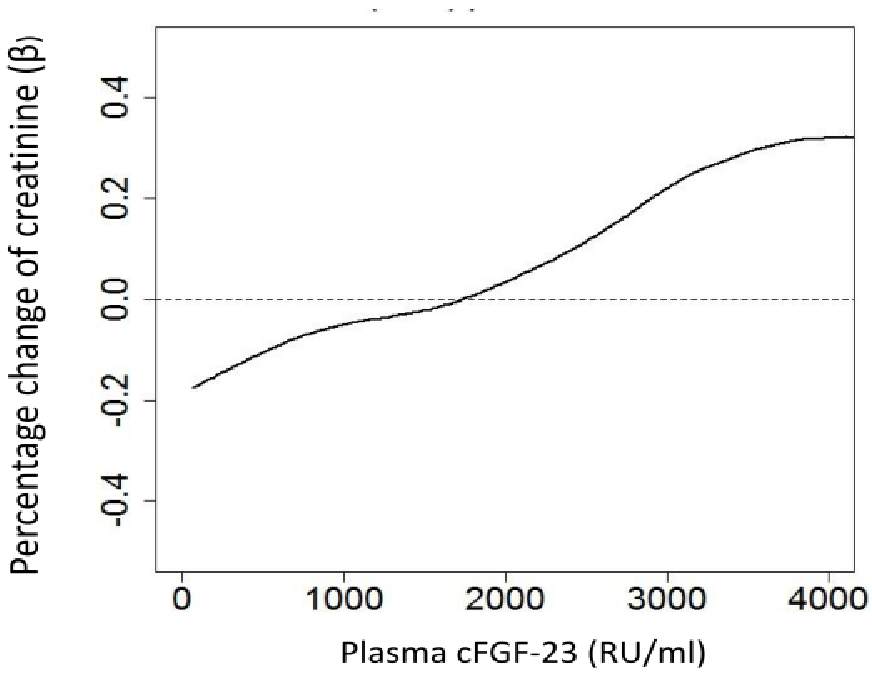
Percentage change of creatinine (β)
0.4
0.2
0.0
-0.2
-0.4
0
1000
2000
3000
4000
Plasma cFGF-23 (RU/ml)

(D)

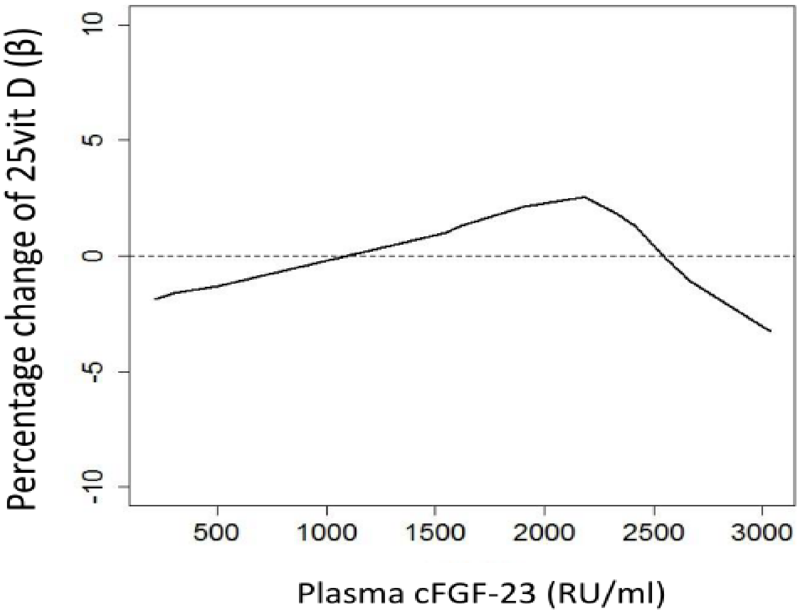
Percentage change of 25vit D (β)
10
5
0
-5
-10
500
1000
1500
2000
2500
3000
Plasma cFGF-23 (RU/ml)

(E)

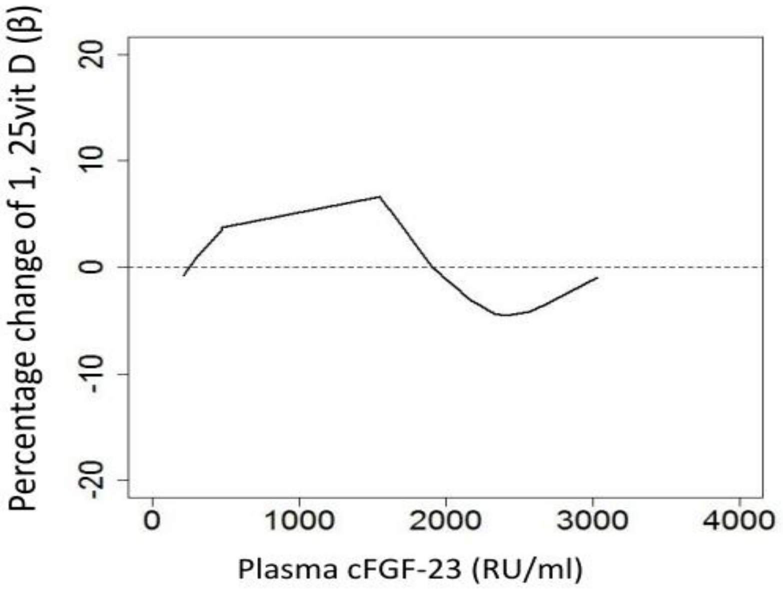
Percentage change of 1, 25vit D (β)
20
10
0
-10
-20
0
1000
2000
3000
4000
Plasma cFGF-23 (RU/ml)

(F)

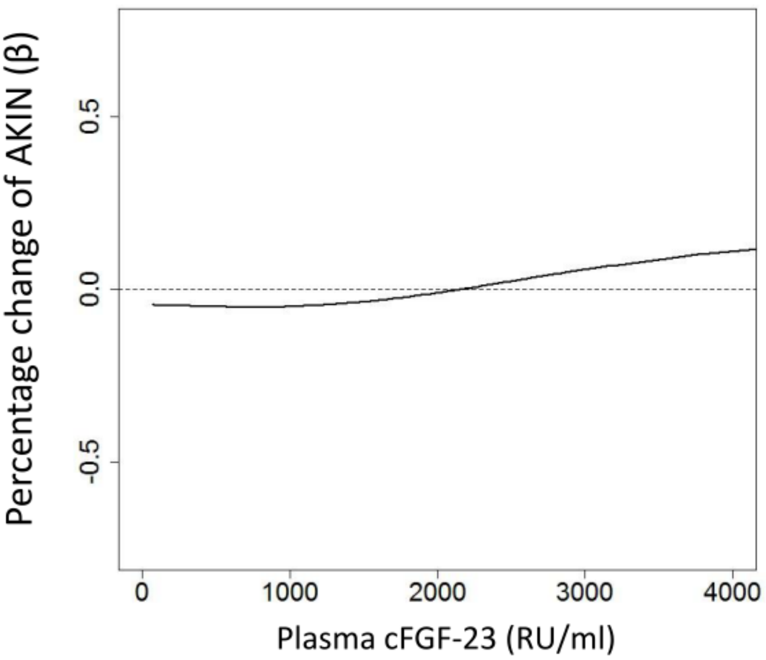
Percentage change of AKIN (β)
0.5
0.0
-0.5
0
1000
2000
3000
4000
Plasma cFGF-23 (RU/ml)

(G)

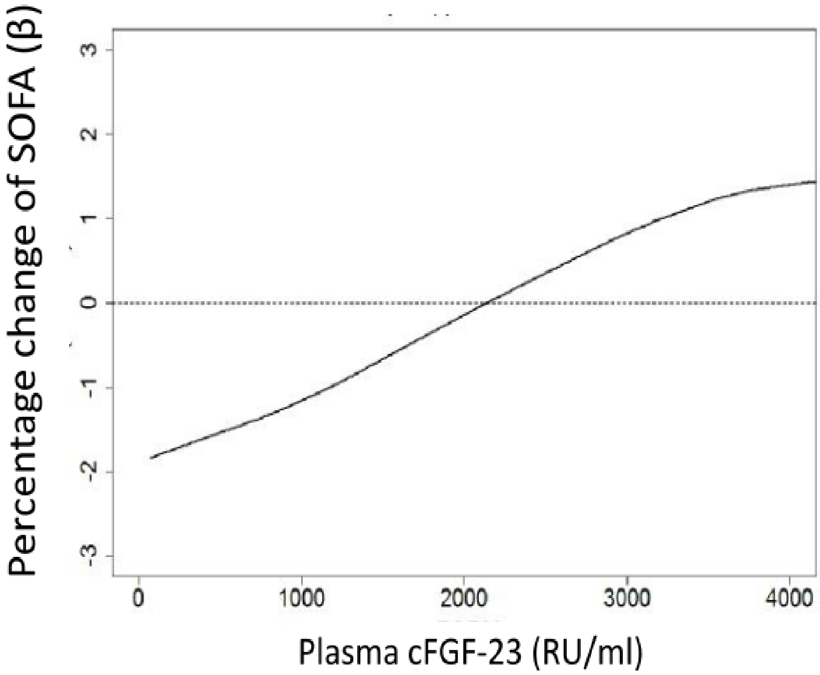
Percentage change of SOFA (β)
3
2
1
0
-1
-2
-3
0
1000
2000
3000
4000
Plasma cFGF-23 (RU/ml)

Supplementary Figure 2.

Decision curve analysis (DCA) plot to assess the clinical consequences of screening AKI-D patients with 90 day mortality using cFGF-23 in addition to AKI risk prediction score (12)**.** Y-axis is the net benefit of the decision strategy. Net benefit is the net proportion of patients with 90 day mortality who would be offered predicting model, without offering predicting model to patients with good outcomes. For patient at AKI at dialysis initialization, forecasting with the AKI risk predicting model with cFGF-23 would yield no net benefit. For risk thresholds between 20 and 80% the superior strategy is forecasting with the AKI risk prediction score with cFGF-23. For moderate to high-risk thresholds (0 to 20%), using the AKI risk prediction score with Sepsis-3 model would yield no net benefit above a predicting none strategy.

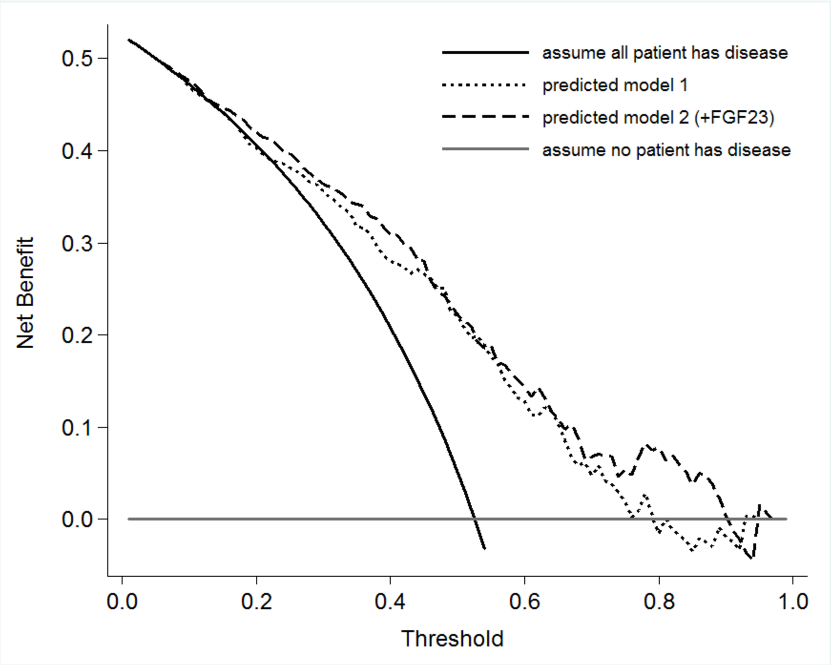

Supplementary Figure 3. The correlation of AKI risk predicting score and AKI risk predicting score with cFGF-23 predicting 90 day mortality. The graph shows 90 day mortality after index hospital discharge predicted by a model containing only the AKI risk predicting score (horizontal axis) against risk predicted by a model containing AKI risk predicting score and cFGF-23 (vertical axis). Lines at predicted risks of 10% and 80% are superimposed to show reclassification over clinically relevant cut points and thereby create a visual representation of a reclassification table. Of note, most limited number of AKI-D patients are reclassified over the cut points (i.e., only a small proportion of dots lies in the off-diagonal cells of the graph). The diagonal line indicates a line of identity; for points above this line, the predicted risk is higher in the new model (improved reclassification for events), and for points below this line, the predicted risk is lower (improved reclassification for non-event cases).

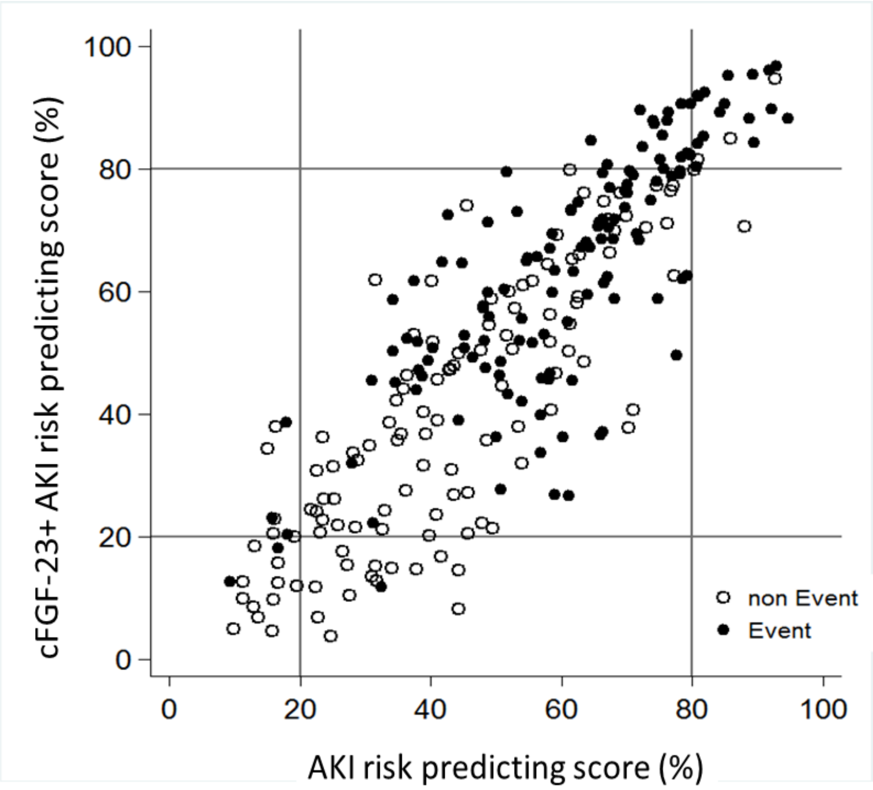
cFGF-23+ AKI risk predicting score (%)
AKI risk predicting score (%)
0
20
40
60
80
100
non Event
Event

Additional Tables

Supplemental Data

Supplemental Data **Table 1. p value comparison of the receiver operating characteristic (ROC) curve for discriminative ability**

	SOFA	cFGF-23	Adjusted NGAL	Adjusted KIM1	AKI risk score	Creatinine
SOFA	NA	0.669	<0.001	0.001	0.450	0.061
cFGF-23		NA	0.001	0.003	0.832	0.168
Adjusted NGAL			NA	0.684	0.003	0.071
Adjusted KIM1				NA	0.009	0.160
AKI risk score					NA	0.168
Creatinine						NA

Abbreviations: Cre, creatinine; cFGF-23, c-terminal fibroblast growth factor-23; KIM-1, Kidney Injury Molecule-1; NGAL, neutrophil

gelatinase-associated lipocalin; SOFA, Sequential Organ Failure Assessment; KDIGO, Kidney Disease Improving Global Outcomes; AKI, acute kidney injury.

Supplemental Data **Table 2. Interaction of baseline co-morbidity with high cFGF-23 predicting 90-day mortality.**

	Interaction with high cFGF-23	***HR (95% CI)***	***p***
SOFA	***0.991***	***0.838-1.172***	***0.991***
AKI risk predicting score	***0.912***	***0.465-1.788***	***0.789***
DM	***0.584***	***0.255-1.336***	***0.584***
Sepsis	***1.027***	***0.442-2.384***	***0.951***
CKD	***1.430***	***0.605-3.379***	***0.415***
Cirrhosis	***0.878***	***0.549-1.403***	***0.878***
BUN at dialysis	***0.992***	***0.984-1.001***	***0.051***
Oliguria at dialysis	***0.571***	***0.266-1.223***	***0.149***

Abbreviations: AKI, acute kidney injury; BUN, blood urea nitrogen; CKD, chronic kidney disease; DM, diabetic mellitus,

References

1. Wu VC, Wang YT, Wang CY, et al. High frequency of linezolid-associated thrombocytopenia and anemia among patients with end-stage renal disease. Clin Infect Dis 2006;42(1):66-72.
2. Knaus WA, Draper EA, Wagner DP, et al. APACHE II: a severity of disease classification system. Crit Care Med 1985;13(10):818-829.
3. Vincent JL, Moreno R, Takala J, et al. The SOFA (Sepsis-related Organ Failure Assessment) score to describe organ dysfunction/failure. On behalf of the Working Group on Sepsis-Related Problems of the European Society of Intensive Care Medicine. Intensive Care Med 1996;22(7):707-710.
4. Chen YS, Ko WJ, Lin FY, et al. Preliminary result of an algorithm to select proper ventricular assist devices for high-risk patients with extracorporeal membrane oxygenation support. J Heart Lung Transplant 2001;20(8):850-857.
5. Wu VC, Ko WJ, Chang HW, et al. Early renal replacement therapy in patients with postoperative acute liver failure associated with acute renal failure: effect on postoperative outcomes. Journal of the American College of Surgeons 2007;205(2):266-276.
6. Lin YF, Ko WJ, Wu VC, et al. A modified sequential organ failure assessment score to predict hospital mortality of postoperative acute renal failure patients requiring renal replacement therapy. Blood Purif 2008;26(6):547-554.
7. Shiao CC, Ko WJ, Wu VC, et al. U-curve association between timing of renal replacement therapy initiation and in-hospital mortality in postoperative acute kidney injury. PloS one 2012;7(8):e42952.
8. Wu VC, Ko WJ, Chang HW, et al. Risk factors of early redialysis after weaning from postoperative acute renal replacement therapy. Intensive Care Med 2008;34(1):101-108.
9. Shiao CC, Wu VC, Li WY, et al. Late initiation of renal replacement therapy is associated with worse outcomes in acute kidney injury after major abdominal surgery. Crit Care 2009;13(5):R171.
10. Leaf DE, Jacob KA, Srivastava A, et al. Fibroblast Growth Factor 23 Levels Associate with AKI and Death in Critical Illness. J Am Soc Nephrol 2016.
11. Vickers AJ, Cronin AM, Elkin EB, et al. Extensions to decision curve analysis, a novel method for evaluating diagnostic tests, prediction models and molecular markers. BMC Med Inform Decis Mak 2008;8:53.

12. http://wwwnhigovtw/webdata/webdataaspx?menu=17&menu_id=1023&WD_ID=1043&webdata_id=4145.
